# Supplementary material for: Flexible and Highly Sensitive Humidity Sensor Based on Sandwich-Like Ag/Fe3O4 Nanowires Composite for Multiple Dynamic Monitoring
Source: Nanomaterials (Basel). 2019 Oct 1;9(10):1399. doi: 10.3390/nano9101399 (PMC6835934; doi:10.3390/nano9101399)
Supplement: Supplementary file 1 [file nanomaterials-09-01399-s001.pdf]

Flexible and Highly Sensitive Humidity Sensor Based on Sandwich-Like Ag/Fe_3O_4 Nanowires Composite for Multiple Dynamic Monitoring

Maojiang Zhang [1,2,3], Minglei Wang [1,2], Mingxing Zhang [1,2], Long Qiu [1], Yinjie Liu [1], Wenli Zhang [1,2], Yumei Zhang [4], Jiangtao Hu [1,*] and Guozhong Wu [1,3,*]

1 CAS Center for Excellence on TMSR Energy System, Shanghai Institute of Applied Physics, Chinese Academy of Sciences, No. 2019 Jialuo Road, Jiading District, Shanghai 201800, China; zhangmaojiang@sinap.ac.cn (M.Z.); wangminglei@sinap.ac.cn (M.W.); zhangmingxing@sinap.ac.cn (M.Z.); qiulong@sinap.ac.cn (L.Q.); liuyinjie@sinap.ac.cn (Y.L.); zhangwenli@sinap.ac.cn (W.Z.); hujiangtao@sinap.ac.cn (J.H.); wuguozhong@sinap.ac.cn (G.W.)

2 School of Nuclear Science and Technology, University of Chinese Academy of Sciences, Beijing 100049, China

3 School of Physical Science and Technology, Shanghai Tech University, Shanghai 200031, China

4 State Key Laboratory for Modification of Chemical Fibers and Polymer Materials, Donghua University, Shanghai 201620, China; zhangym@dhu.edu.cn (Y.Z.)

* Correspondence: hujiangtao@sinap.ac.cn (J.H.); wuguozhong@sinap.ac.cn (G.W.); Tel.: 0000-0002-0210-9603 (J.H.); 0000-0003-3814-2074 (G.W)

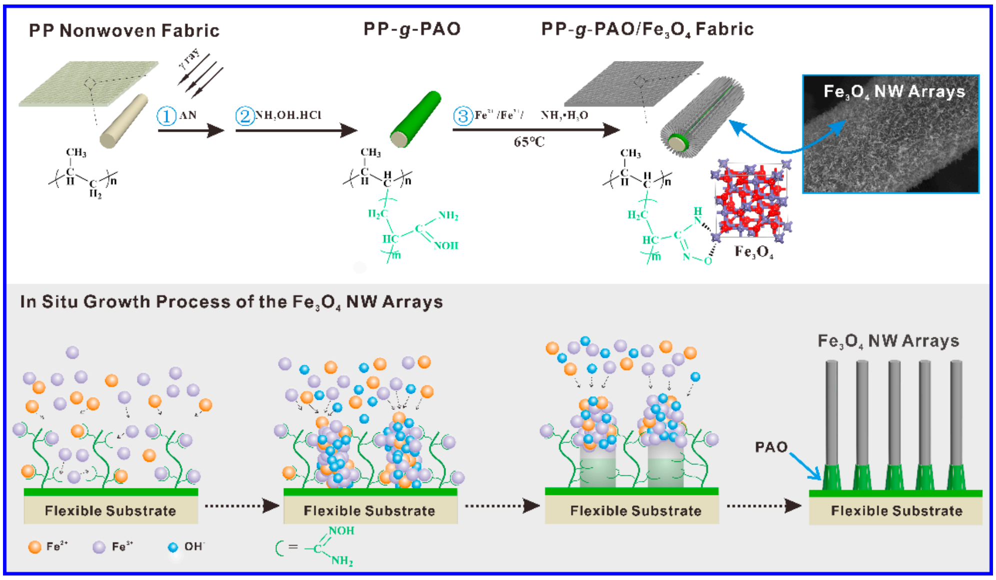

Figure S1. Schematic of the growth process of Fe_3O_4 NW arrays on the surface of PP nonwoven fabric and the chemical structure of the PP-*g*-PAO/Fe_3O_4 composite.[1]

Preparation of the amidoxime PP nonwoven fabrics: Before graft polymerization, the PP nonwoven fabric was extracted with boiling acetone over 12 h to remove the impurities attached on the surface. Then it was placed in tubes containing a 20:80 vol% AN:DMF solution. The solution was degassed by bubbling with nitrogen for 20 min to remove the oxygen, and the tubes were then sealed and irradiated with a ^{60}Co γ-ray source for 17 h at room temperature. The total absorbed dose was 20 kGy. The grafted PP nonwoven fabric (coded as PP-*g*-PAN) was washed using DMSO and ethanol to remove the residual monomer and homopolymer, and then dried in a vacuum oven at 60 °C to a constant weight. The degree of grafting (DG) was

determined according to Equation (1):

$$DG(\%) = \frac{(W_1 - W_0)}{W_0} \times 100\% \qquad (1)$$

where W_0, W_1 are the weights of PP nonwoven fabrics before and after grafting, respectively. In this work, the DG of PP-*g*-PAN was 46.4%.

The PP-*g*-PAN fabric was reacted with hydroxylamine hydrochloride in a DMSO/H_2O (v/v = 1:1) solution with pH 7.0 at 80 °C for 4 h. The molar ratio of the cyano group to hydroxylamine hydrochloride was 1:8. Subsequently, the sample was removed from the solution and repeatedly washed with distilled water to remove any residual reagent, and then dried in a vacuum oven at 60 °C. The resultant material was designated as PP-*g*-PAO.

Preparation of PP-*g*-PAO/Fe_3O_4 composite nonwoven fabrics: In a three-necked flask, $FeCl_3 \cdot 6H_2O$ (10.8 g) and $FeSO_4 \cdot 7H_2O$ (6.7 g) were dissolved in deionized water (200 ml), and then the PP-*g*-PAO (0.5 g) was added with continuous stirring for 30 min at 65 °C under N_2 atmosphere. A diluted ammonia solution (3.8-4.2 wt%, 100 mL) was added dropwise into the flask over 60 min and kept at 65 °C for 3 h under continuous stirring. The obtained composite was ultrasonically cleaned for 40 min to remove Fe_3O_4 nanoparticles (NPs) adsorbed on the surface of the PP-*g*-PAO/Fe_3O_4 *via* Van der Waals forces. Finally, the sample was repeatedly washed with distilled water until the pH reached 7, and then dried in a vacuum oven at 60 °C. The resultant material was designated as PP-*g*-PAO/Fe_3O_4.

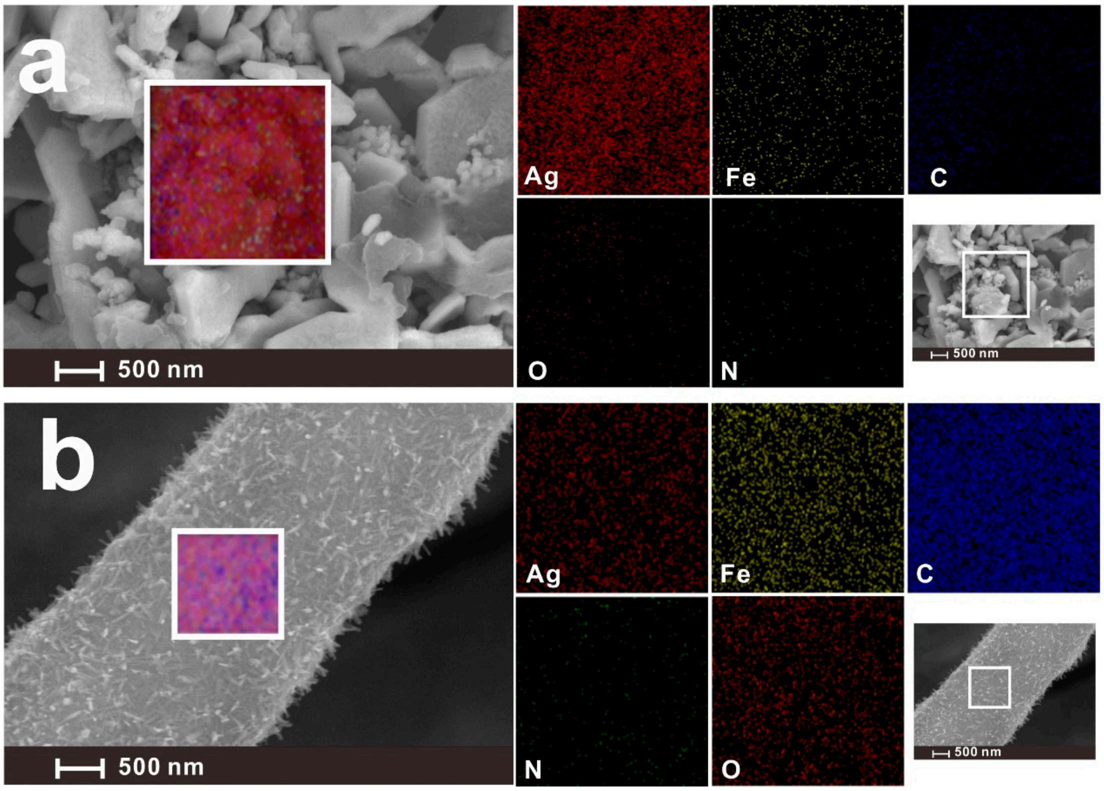

Figure S2. EDS mappings of Ag, Fe, C, O and N on the Ag@PP-*g*-PAO/Fe_3O_4 fabrics corresponding to the SEM images: (a) the outer surface and (b) the inner surface of Ag@PP-*g*-PAO/Fe_3O_4 based sensor.

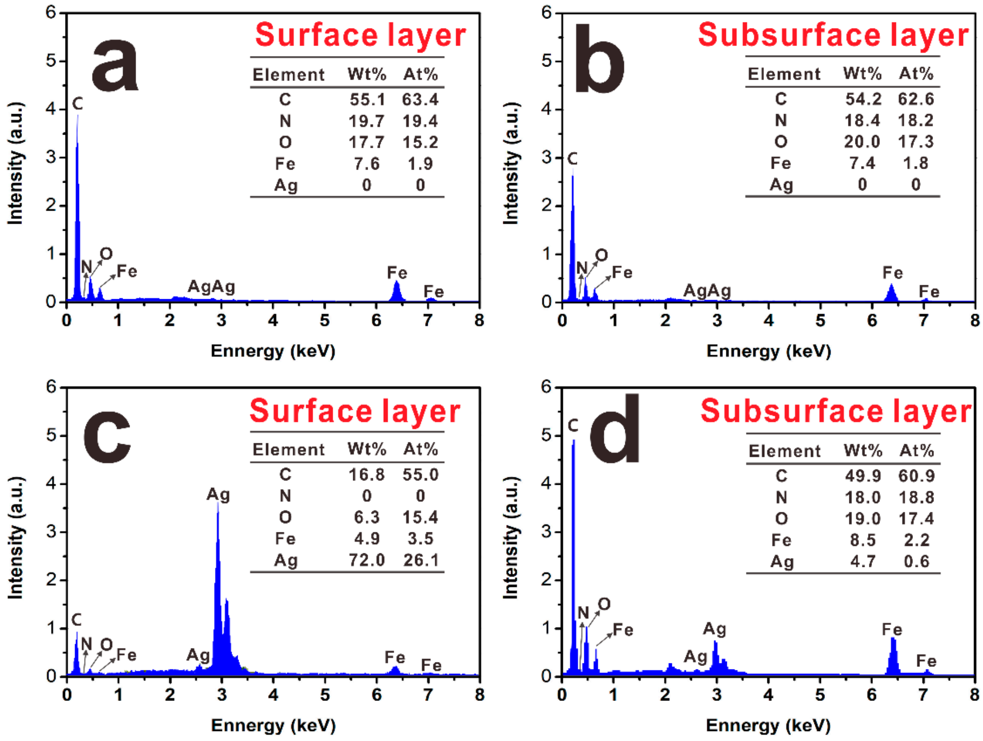

Figure S3. EDS spectra of (**a**,**b**) PP-*g*-PAO/Fe_3O_4 and (**c**,**d**) Ag@Fe_3O_4-MS.

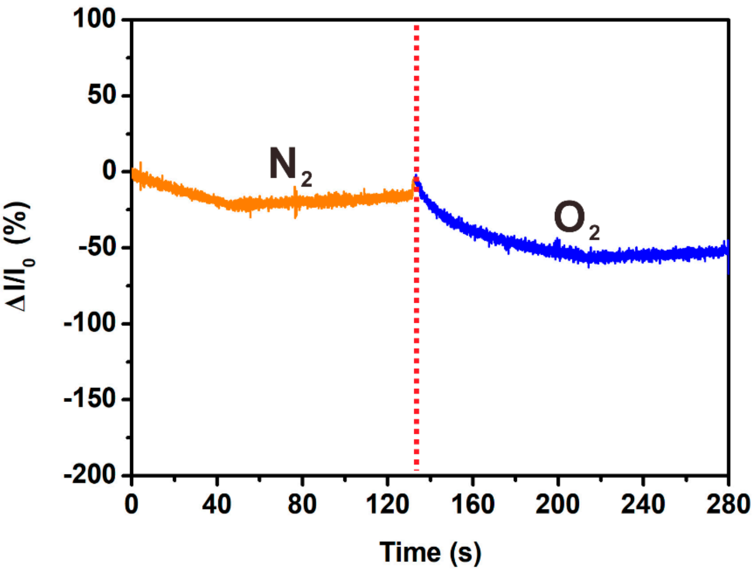

Figure S4. The influence of Ag@Fe_3O_4-MS to pure nitrogen, and pure oxygen.

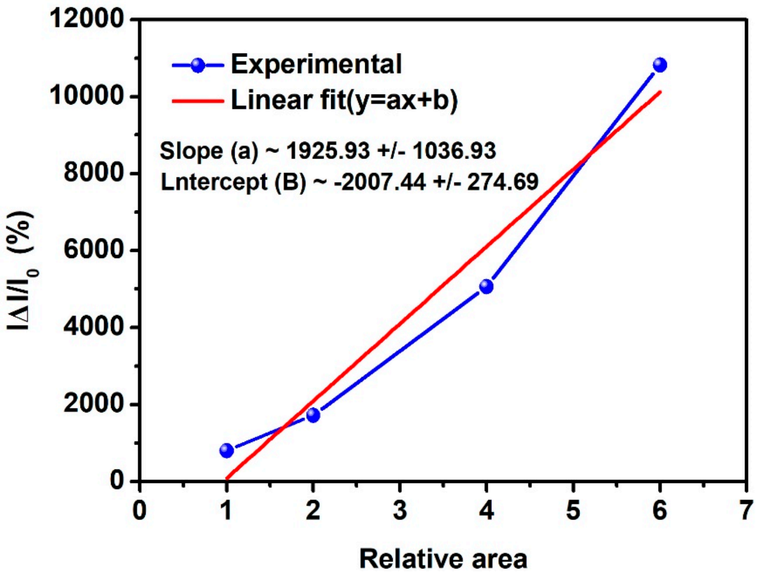

Figure S5. Relative current intensity for Ag@Fe_3O_4-MS versus relative areas.(Relative area is defined as the ratio of sample area to minimum sample area.)

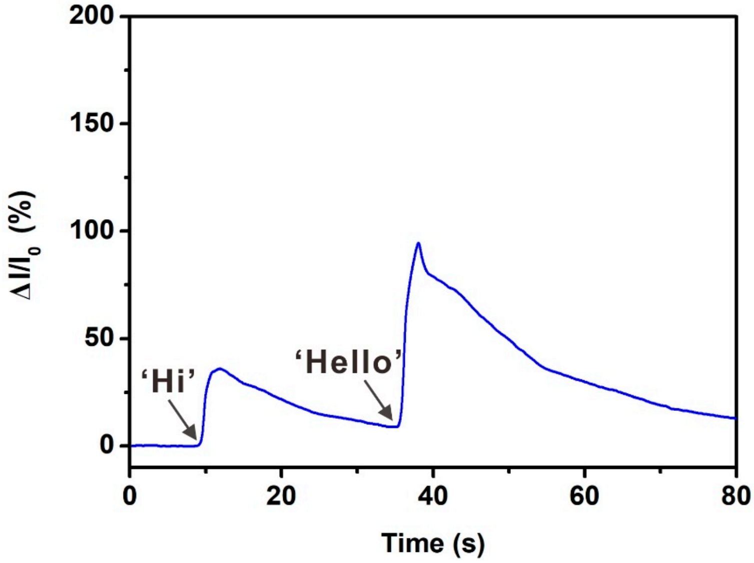

Figure S6. Current responses of the sensor sewn into a face mask when the wearer said the words "hi" and "hello", showing the ability to distinguish different sounds.

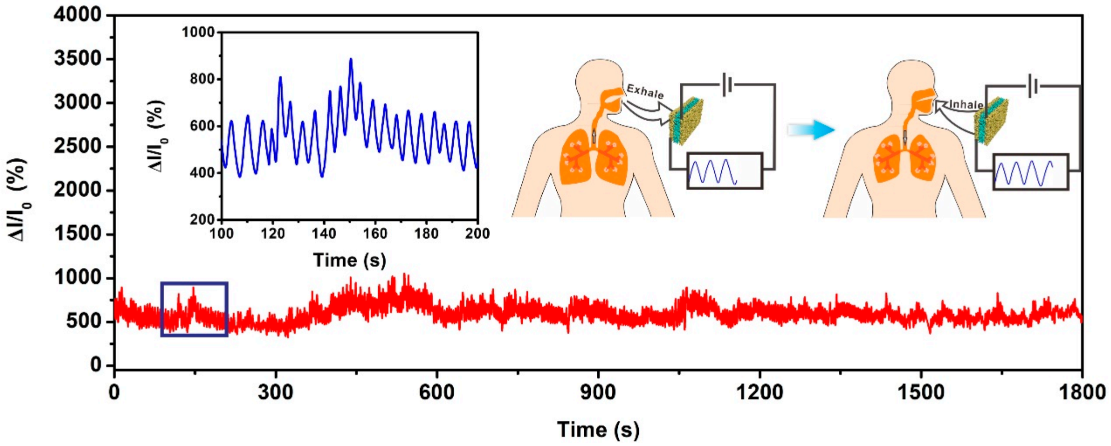

Figure S7. Detection of the rate and strength of adult respiration for 30 min. Inset on the left: enlarged view of the breathing frequency plot. Insets on the right: the working mechanism of the Ag@Fe_3O_4-MS sensor for detecting respiration.

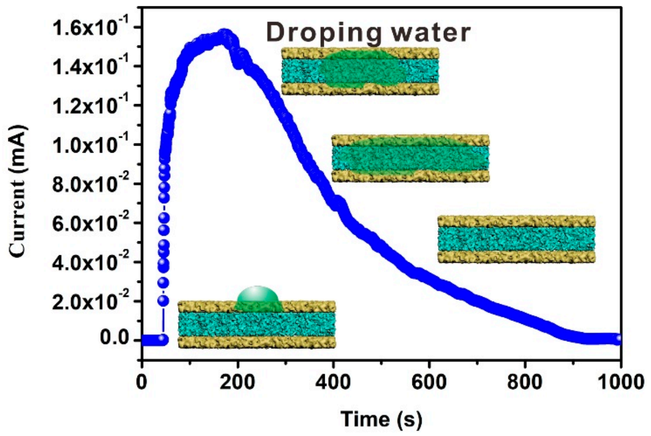

Figure S8. Ag@Fe_3O_4-MS sensor responses to a water droplet placed on it.

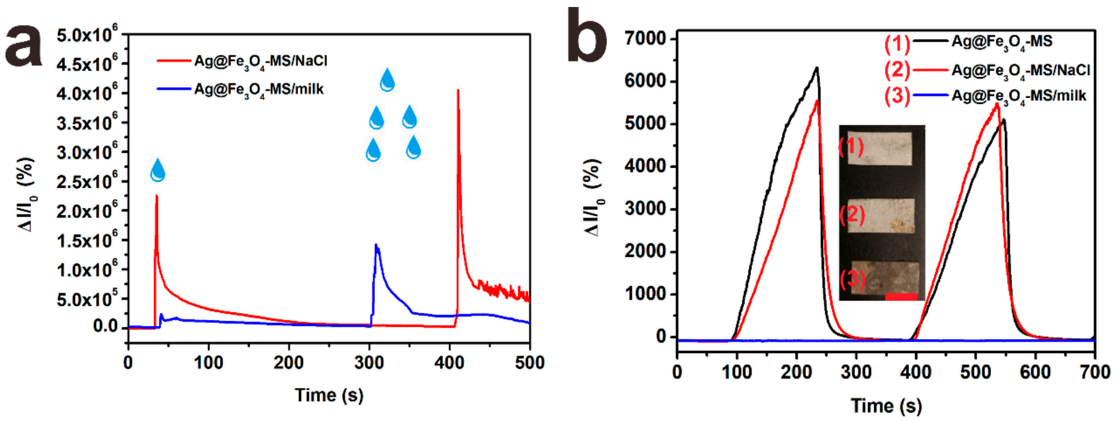

Figure S9. (**a**) Ag@Fe_3O_4-MS sensor responses to a NaCl solution (or milk) droplet placed on it, and then five NaCl solution (or milk) droplets; NaCl solution is 1 g/L. (**b**) Dependence of relative current of Ag@Fe_3O_4-MS at RH = 95%, before and after the sensor was assessed in additional NaCl solution or milk; insert of corresponding photographs. Scale: 10 mm.

References

[1] M. Zhang, M. Wang, M. Zhang, A. Maimaitiming, L. Pang, Y. Liang, J. Hu, G. Wu, Fe_3O_4 nanowire arrays on flexible polypropylene substrates for UV and magnetic sensing, ACS Appl. Nano Mater. 1(10) (2018) 5742-5752.
